# Infrared Target Reconstruction Under Detector Multiplexing Using Polarization Encoding and Stokes Vector Decoding

**DOI:** 10.3390/s26082286

**Published:** 2026-04-08

**Authors:** Menghan Bai, Zibo Yu, Guanyu Mu, Zhenyuan Guo, Chunyu Liu

**Affiliations:** 1 Changchun Institute of Optics, Fine Mechanics and Physics, Chinese Academy of Sciences, Changchun 130033, China; baimenghan21@mails.ucas.ac.cn (M.B.); muguanyu23@mails.ucas.ac.cn (G.M.); guozhenyuan23@mails.ucas.ac.cn (Z.G.); 2 University of Chinese Academy of Sciences, Beijing 101408, China

**Keywords:** polarization encoding, infrared imaging, detector multiplexing, stokes vector reconstruction, computational imaging

## Abstract

Wide-field infrared imaging systems are often constrained by detector size, cooling requirements, and payload limitations, leading to the need for multi-FOV detector sharing. However, conventional geometric multiplexing introduces severe spatial aliasing, which significantly degrades target localization performance. This paper proposes a polarization-encoded field-of-view multiplexing method for recovering spatial information from aliased detector measurements. The imaging plane is divided into multiple FOV regions, each assigned a distinct polarization state. After optical folding, the modulated sub-images are superimposed onto a common detector region. Six-channel polarization measurements are used to reconstruct pixel-wise Stokes vectors, and the spatial origin of each pixel is identified through polarization-domain similarity matching and target-level voting. MATLAB-based simulations were conducted using a nine-region multiplexing configuration. The proposed method achieves 97.3% pixel-level classification accuracy under ideal conditions and maintains over 95% accuracy at a noise level of σ = 0.02. The normalized Stokes reconstruction error is below 0.02, and stable performance is observed under polarization modulation deviations within ±10°. By introducing polarization as an additional encoding dimension, the proposed framework enables efficient separation of multiplexed spatial information without increasing detector resources, demonstrating its potential for compact wide-field infrared sensing applications.

## 1. Introduction

Wide-field infrared detection systems play a crucial role in numerous applications, including space situational awareness, missile early warning, and long-range target monitoring [1,2,3]. These systems are typically required to simultaneously achieve large field-of-view (FOV) coverage and high detection sensitivity. However, due to strict constraints on detector size, weight, power consumption, and cost, modern infrared sensing platforms often rely on small-format focal plane arrays (FPAs) [4]. As a result, detector resource limitations have become a fundamental bottleneck restricting system performance, particularly when wide-area surveillance and high-resolution detection must be realized simultaneously.

To overcome detector limitations, multiplexing imaging architectures have attracted significant research attention [5,6,7]. By mapping signals from multiple FOV regions onto a single detector, detector reuse can be achieved while maintaining wide-area observation capability. Nevertheless, detector multiplexing inevitably introduces aliasing among signals originating from different spatial regions, which leads to severe degradation in target detectability and localization accuracy [8]. Therefore, developing effective encoding and decoding strategies capable of separating multiplexed signals has become a key challenge in advanced infrared sensing system design.

Traditional small-target detection algorithms mainly rely on intensity-based or spatial-contrast-based features [9,10,11], including top-hat filtering, local contrast enhancement, and statistical background suppression techniques [12]. Although these methods are effective for conventional imaging systems, they are generally insufficient for multiplexed sensing scenarios where targets originating from different spatial regions may exhibit similar intensity and motion characteristics. Recently, computational imaging approaches have been introduced to address this limitation by embedding physical modulation mechanisms into optical systems, thereby transforming spatial information into discriminative signal features that can be computationally decoded [11,13,14,15].

In our previous work, spatially variant point spread function (PSF) modulation was proposed to encode FOV-dependent information into target spot morphology [11]. By deliberately introducing FOV-dependent spot deformation patterns, aliasing signals could be separated through morphology-aware reconstruction algorithms. This strategy demonstrated the feasibility of physically encoded computational sensing and provided a promising solution for detector reuse in wide-field infrared systems. However, spatial PSF modulation inherently relies on geometric distortion and optical aberration engineering, which limits the modulation dimensionality and may introduce additional sensitivity to manufacturing tolerances and system alignment errors. Furthermore, spatial-domain encoding primarily modulates intensity distribution, restricting the available information channel capacity for signal discrimination.

Polarization, as an intrinsic physical property of electromagnetic waves, provides an additional and largely independent information dimension that has not been fully exploited in detector multiplexing imaging systems [16,17,18,19]. Unlike spatial modulation, polarization modulation enables vector-field encoding of incident radiation, allowing signals from different FOV regions to be mapped into distinct polarization states [20]. By integrating polarization-sensitive optical components and polarization-resolved detection, it becomes possible to construct orthogonal encoding channels without significantly increasing system complexity or sacrificing optical throughput. This capability offers a new pathway to enhance information capacity in multiplexed sensing systems.

Motivated by these advantages, this paper proposes a polarization-domain computational imaging framework for wide-field infrared detector multiplexing systems. A FOV-dependent polarization encoding scheme is introduced by modulating the polarization states of incident radiation at the intermediate image plane. Subsequently, polarization-resolved detection is performed to extract Stokes vector parameters, which are used to reconstruct multiplexed target signals through a vector-based decoding algorithm. Compared with conventional spatial modulation methods, the proposed approach extends the modulation domain from scalar intensity encoding to vector-field polarization encoding, significantly improving signal separability and reconstruction robustness.

This study establishes a unified physical and computational model describing the polarization encoding, optical folding, and polarization-domain decoding processes. The proposed framework provides a new pathway for detector resource reutilization in compact infrared sensing platforms. It also demonstrates the potential of polarization as an additional information dimension for computational imaging. The effectiveness of the method is validated through systematic simulations and analyses, highlighting its capability to improve target discrimination and reconstruction accuracy in multiplexed wide-field imaging scenarios. It is worth noting that polarization multiplexing has been previously explored for field-of-view extension in optical systems [21]. However, unlike conventional approaches that utilize polarization for parallel imaging, the proposed method addresses a fundamentally different problem, namely the reconstruction of spatial information under severe detector multiplexing-induced aliasing. In this work, polarization is not used to separate optical paths, but rather to encode field-of-view-dependent information into the Stokes domain, which is subsequently decoded through computational reconstruction. This forward–inverse modeling framework distinguishes the proposed method from existing polarization multiplexing techniques and establishes a new paradigm for detector-sharing infrared imaging systems.

## 2. Polarization Encoding Mechanism

To enable reliable target reconstruction under detector multiplexing conditions, it is necessary to introduce an additional physical encoding dimension that can distinguish signals originating from different field-of-view (FOV) regions after optical superposition. Polarization provides a naturally orthogonal information channel that can be modulated independently from spatial intensity distribution while remaining physically compatible with compact optical systems.

**Figure 1:**
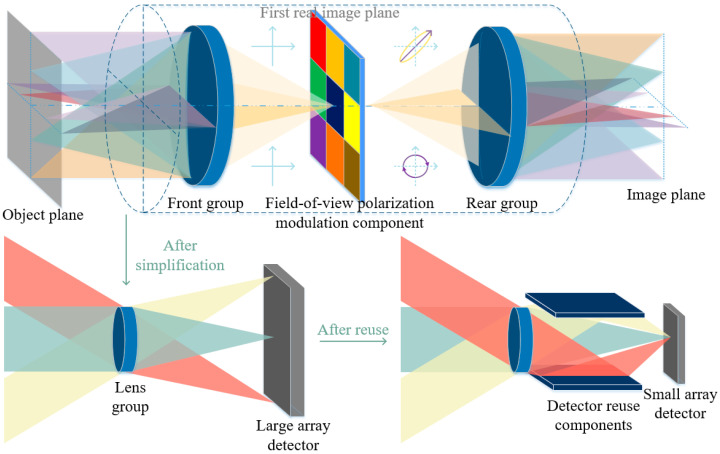
Schematic diagram of the polarization-encoded field-of-view (FOV) multiplexing framework. (**Top**) The physical encoding process, where incident radiation from different FOVs is modulated by a polarization mask and then imaged. The polarization modulation is implemented at the intermediate image plane, where spatially separated FOV regions are directly encoded with distinct polarization states. (**Bottom Left**) The simplified optical model showing spatial aliasing when using a large-format detector without multiplexing logic. (**Bottom Right**) The proposed detector reuse architecture, where multi-FOV signals are folded and superimposed onto a small-array detector, with polarization states serving as the discriminative tag for spatial reconstruction.

In this work, as shown in Figure 1, a polarization encoding strategy is developed to map spatial FOV information into distinct polarization states. By assigning each sub-FOV region a unique polarization modulation mode, spatial aliasing introduced by optical folding can be transformed into polarization-domain multiplexing. This transformation allows the overlapped signals to remain distinguishable through polarimetric measurements and Stokes vector reconstruction.

This section first establishes the theoretical framework of polarization-encoded FOV multiplexing and then introduces the mathematical representation of polarization states based on Stokes parameters. The polarization state design principles and encoding strategy for multi-region FOV discrimination are subsequently presented.

### 2.1. Polarization-Encoded Field-of-View Multiplexing Framework

In space-based wide-field infrared detection systems, detector arrays are often constrained by cooling requirements, pixel fabrication complexity, and system payload limitations. Consequently, a single focal plane array (FPA) is frequently required to receive optical signals from multiple spatial field-of-view (FOV) regions simultaneously. Conventional detector multiplexing techniques rely on geometric optical folding or spatial sampling compression, which inevitably leads to severe aliasing among different FOV sub-images, thereby degrading target detection and localization accuracy.

To overcome this limitation, this work proposes a polarization-encoded FOV multiplexing framework, in which spatial FOV information is mapped into polarization states. The encoded sub-images are geometrically folded and overlapped onto a single detector sub-region, and the original spatial information is reconstructed through Stokes vector inversion and polarization matching.

Let the full imaging plane be divided into K independent FOV regions:

(1) $$\Omega =\overset{K}{\underset{k=1}{⋃}}\Omega_{k}$$

Each region is assigned a unique polarization modulation state. After optical folding, the detector receives a superimposed signal:

(2) $$I_{mix}(x,y)=\sum_{k=1}^{K}I_{k}^{\prime }(x,y)$$

where $I_{k}^{\prime }(x,y)$ represents the geometrically transformed intensity distribution of the k-th FOV region.

Compared with spatial or morphological encoding methods, polarization encoding introduces an orthogonal physical dimension that enables robust discrimination of aliased sub-images while maintaining system compactness.

### 2.2. Polarization Encoding Model and Stokes Representation

The polarization state of the optical field in each FOV region is characterized using the Stokes vector [22,23,24]:

(3) $$S=\left[\begin{array}{c} S_{0}\\ S_{1}\\ S_{2}\\ S_{3} \end{array}\right]$$

where $S_{0}$ denotes the total intensity, and $S_{1},S_{2},S_{3}$ represent the linear and circular polarization components.

For fully polarized light, the Stokes parameters can be parameterized by polarization azimuth angle ψ and ellipticity angle χ:

(4) $$\begin{array}{cc} S_{1} & =S_{0}cos\;(2\psi )cos\;(2\chi )\\ S_{2} & =S_{0}sin\;(2\psi )cos\;(2\chi )\\ S_{3} & =S_{0}\sin\;(2\chi ) \end{array}$$

In this study, the full FOV is partitioned into nine sub-regions, each assigned a distinct polarization state including linear, circular, and elliptical polarization modes. This configuration provides sufficient polarization diversity and ensures distinguishability after aliasing.

To ensure robust discrimination among multiplexed FOV regions, the polarization states are designed to span the Stokes space with maximal angular separation. The adopted configuration includes four linear polarization states (0°, 45°, 90°, 135°), two circular polarization states (right- and left-handed), and three elliptical polarization states with different azimuth and ellipticity angles. This arrangement provides a near-uniform distribution in the Stokes space, minimizing inter-channel correlation and improving classification robustness under noise and modulation errors.

## 3. Polarization-Domain Demultiplexing and Target Reconstruction Method

### 3.1. Optical Folding Aliasing and Polarization Measurement Model

To achieve detector multiplexing, the encoded sub-images are folded by reflective optical elements. The folding process is mathematically described as a spatial transformation operator:

(5) $$I_{k}^{\prime }(x,y)=T_{k}\left(I_{k}(x,y)\right)$$

where $T_{k}(\cdot )$ represents reflection, rotation, or diagonal folding operations corresponding to different FOV regions.

The detector integrates the optical energy from all folded sub-images, forming an aliased intensity distribution. To retrieve polarization information from the overlapped image, multiple polarization analyzer measurements are performed [25].

The measurement intensity under a linear analyzer oriented at angle θ is expressed as:

(6) $$I(\theta )=\frac{1}{2}\left(S_{0}+S_{1}cos\;2\theta +S_{2}sin\;2\theta \right)$$

The circular polarization components are obtained using right-hand and left-hand analyzers:

(7) $$I_{R}=\frac{1}{2}(S_{0}+S_{3}),I_{L}=\frac{1}{2}(S_{0}-S_{3})$$

These multi-channel measurements form a polarization observation vector that enables recovery of the Stokes parameters from aliased data.

In practical implementations, the acquisition of the full Stokes vector requires multiple polarization measurements, including both linear and circular components. A conventional approach employs a rotating linear polarizer combined with a quarter-wave plate to sequentially capture different polarization states. While this method is straightforward, it introduces temporal overhead and may reduce the effective frame rate in dynamic imaging scenarios.

To overcome this limitation, snapshot polarization imaging techniques can be adopted. In particular, division-of-focal-plane (DoFP) polarization cameras enable simultaneous acquisition of multiple polarization components within a single exposure by integrating micro-polarizer arrays at the detector level. Such architectures allow parallel measurement of polarization information without mechanical modulation.

Therefore, the proposed polarization-encoded multiplexing framework is compatible with both sequential and snapshot polarization measurement schemes, providing flexibility for different application requirements.

### 3.2. Stokes Reconstruction and Polarization-Based FOV Decoding

Based on the six polarization measurement channels, the Stokes vector is reconstructed through linear inversion:

(8) $$\begin{array}{cc} S_{0} & =I_{0^{\circ }}+I_{90^{\circ }}\\ S_{1} & =I_{0^{\circ }}-I_{90^{\circ }}\\ S_{2} & =I_{45^{\circ }}-I_{135^{\circ }}\\ S_{3} & =I_{R}-I_{L} \end{array}$$

The degree of linear polarization and circular polarization are further derived as:

(9) $$DoLP=\frac{\sqrt{S_{1}^{2}+S_{2}^{2}}}{S_{0}},DoCP=\frac{|S_{3}|}{S_{0}}$$

To identify the spatial origin of each aliased pixel, the reconstructed Stokes vector is compared with predefined polarization reference vectors corresponding to each FOV region. The classification is performed using normalized vector similarity:

(10) $$\rho_{k}=\frac{S\cdot S_{k}}{∥S∥}$$

The pixel is assigned to the FOV region with the highest similarity score. Subsequently, foreground segmentation and majority voting are applied to determine the most probable source region of the detected target, thereby achieving target reconstruction under detector multiplexing conditions.

For clarity, two levels of spatial identification are defined in this study. Pixel-level field-of-view (FOV) classification refers to assigning each detector pixel to its originating FOV region based on the similarity between the reconstructed Stokes vector and predefined polarization reference states. Target-level FOV identification is performed by applying a majority-voting strategy over all pixels belonging to a segmented target region, thereby determining the global FOV origin of the target. This hierarchical definition ensures both fine-grained spatial discrimination and robust target-level localization.

## 4. Simulation Results and Performance Evaluation

### 4.1. Simulation Environment

**Figure 2:**
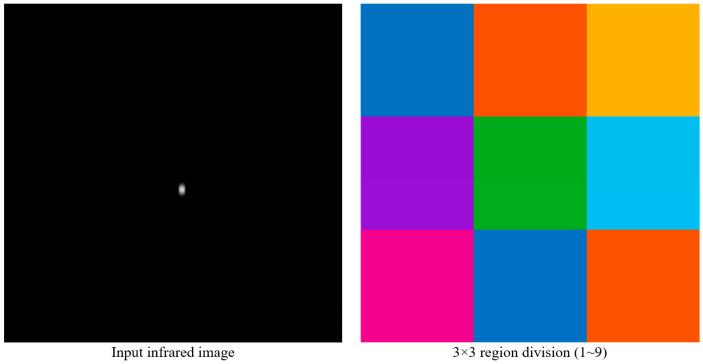
Spatial partition of the full imaging plane into a 3 × 3 grid representing nine independent FOV regions. Each region is assigned a distinct polarization modulation state (linear, circular, or elliptical) to enable polarization-domain encoding prior to optical folding and multiplexing.

To validate the effectiveness of the proposed polarization-encoded FOV multiplexing method, a numerical simulation platform was developed using MATLAB 2024a. As shown in Figure 2, the input infrared image was divided into a 3 × 3 grid representing nine independent FOV regions. Each region was assigned a distinct polarization modulation state including linear, circular, and elliptical polarization modes.

It should be noted that realistic infrared detection scenarios involve complex background radiation and atmospheric effects. In our previous work [11], a detailed radiometric modeling framework was established, incorporating Earth background radiation, atmospheric transmission, and target emission characteristics in the 0.9–2.1 μm band. The results showed that, under typical space-based observation conditions, the background radiation power is on the order of $10^{-15}$ W, which is significantly lower than the radiation from high-temperature targets (~$10^{-6}$ W). This implies that, even under multiplexing conditions, the signal-to-background ratio remains sufficiently high for reliable target detection.

The Stokes parameters of each region were generated based on the theoretical polarization model described in Section 2. Optical folding and aliasing were simulated by superimposing the modulated sub-images. Six-channel polarization measurements were synthesized to reconstruct Stokes vectors.

In this study, a simplified simulation environment is adopted to focus on validating the polarization encoding and reconstruction mechanism. The influence of complex background clutter is therefore not explicitly modeled, but its impact has been analyzed in our previous work [11] and is discussed in Section 5. The simulated imaging scenario adopts a high-contrast configuration with bright targets and relatively dark backgrounds, which is consistent with typical infrared search and track (IRST) applications where high-temperature targets exhibit significantly stronger radiation than the Earth background. This configuration is supported by radiometric analysis, indicating a several-orders-of-magnitude difference in radiative power. For scenarios involving higher background radiation levels, the proposed polarization encoding mechanism remains applicable, as it relies on polarization-domain discrimination rather than intensity contrast alone.

### 4.2. Polarization Encoding Aliasing and Stokes Reconstruction Consistency Verification

To validate the effectiveness of the proposed polarization-encoded multiplexing framework, both the aliasing formation process and the accuracy of subsequent Stokes reconstruction were jointly analyzed. This evaluation aims to verify whether polarization modulation can preserve distinguishable physical signatures after geometric folding and intensity superposition.

First, polarization encoding was applied to nine predefined FOV sub-regions, where each region was assigned a unique polarization modulation state. After spatial folding through reflective optical elements, the encoded sub-images were geometrically transformed and overlapped onto the same detector region. The resulting detector measurement represents the incoherent superposition of polarization-modulated intensities from all FOV channels.

**Figure 3:**
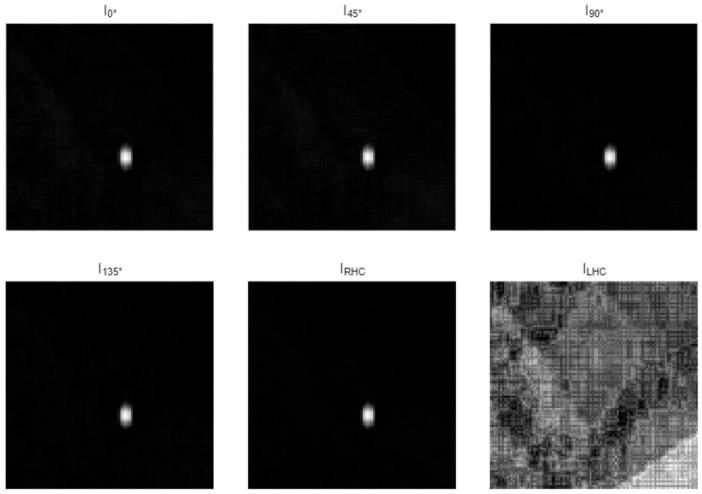
Six-channel polarization intensity measurements after geometric folding and incoherent energy superposition of all nine FOV regions. The displayed frames correspond to linear polarization analyzers at 0°, 45°, 90°, and 135°, as well as right- and left-handed circular polarization components. Severe spatial aliasing is observed, where individual FOV regions cannot be separated using intensity information alone.

Simulation results confirm that severe spatial aliasing occurs in the folded detector image, where individual sub-images cannot be separated through conventional intensity-based observation. However, due to the orthogonality introduced by polarization modulation, each overlapped pixel still retains distinguishable polarization characteristics associated with its original FOV region. The measured intensity distributions of the six polarization channels after geometric folding are shown in Figure 3.

To retrieve the embedded polarization information, multi-channel polarization measurements were performed using six analyzer configurations, corresponding to linear polarization orientations of 0°, 45°, 90°, and 135°, as well as right-handed and left-handed circular polarization states. These measurements form a complete observation basis for reconstructing the Stokes parameters at each pixel location.

**Figure 4:**
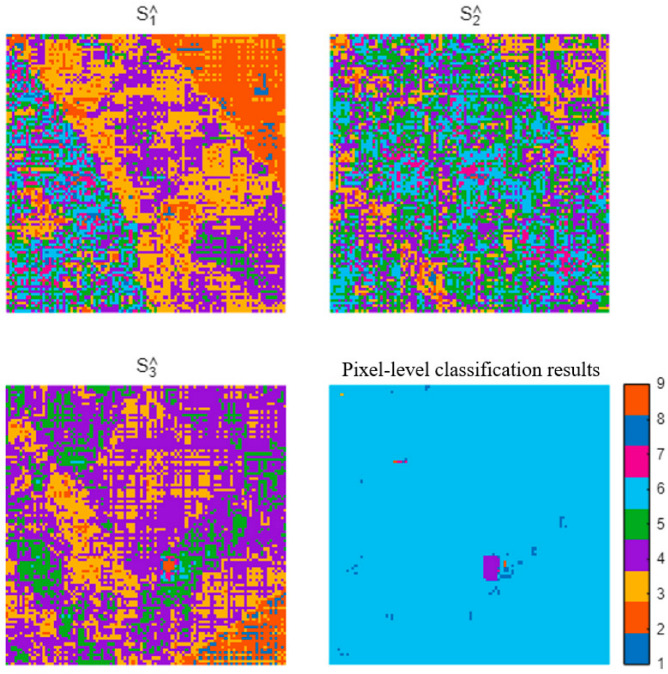
Reconstructed Stokes parameters (S1^, S2^, S3^) obtained from six-channel polarization measurements, along with the pixel-level FOV classification map based on normalized polarization similarity matching. Despite severe spatial overlap in the intensity domain, polarization-domain reconstruction enables effective separation of multiplexed FOV signals.

The reconstructed Stokes vectors were then compared with ground-truth polarization templates assigned during encoding. Quantitative error analysis was conducted by calculating parameter deviations across the full detector plane. The results demonstrate that the reconstructed Stokes parameters exhibit high consistency with the predefined modulation states, indicating that polarization information remains stable after optical folding and aliasing superposition. The reconstructed Stokes parameter maps (S_1_, S_2_, S_3_) and the pixel-level FOV classification results are displayed in Figure 4.

Furthermore, spatial distribution maps of polarization parameters reveal that distinct FOV regions maintain clear separability in the Stokes domain, even when spatial intensity features are completely overlapped. This observation verifies that polarization modulation effectively introduces an additional encoding dimension, enabling reliable discrimination of multiplexed spatial information.

Overall, the joint verification results confirm the physical consistency between polarization encoding and Stokes-based reconstruction. The proposed framework successfully preserves FOV-dependent polarization signatures throughout the multiplexing process, thereby providing a solid foundation for subsequent target classification and reconstruction.

### 4.3. Target Reconstruction Accuracy and Robustness Evaluation

To quantitatively evaluate the performance of the proposed polarization-encoded FOV multiplexing framework, comprehensive experiments were conducted to assess reconstruction accuracy, spatial separability, and robustness against measurement perturbations.

**Figure 5:**
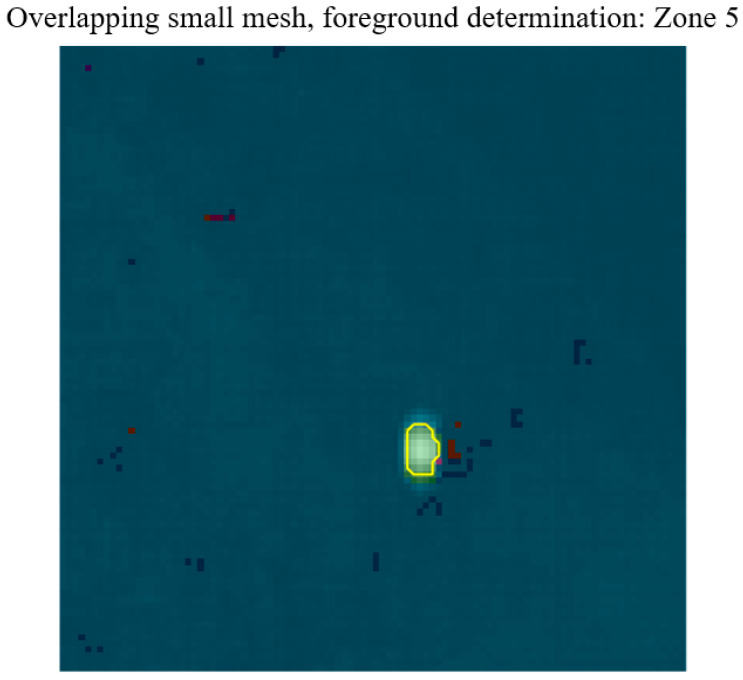
Target-level FOV identification result within the aliased detector region. The reconstructed total intensity map (S0^) is overlaid with pixel-wise classification results. The automatically segmented foreground region is highlighted by yellow boundaries. A majority-voting strategy is applied to determine the global FOV origin of the detected target.

First, pixel-level FOV classification was performed by comparing reconstructed Stokes vectors with predefined polarization templates. Foreground regions were extracted using intensity-based segmentation, and a majority-voting strategy was applied to determine the global FOV origin of each target. The target-level FOV identification result within the aliased detector region is shown in Figure 5. A total of 1000 target instances were simulated at different spatial positions across the nine FOV regions.

Under noise-free conditions, the proposed method achieved a pixel-level classification accuracy of 97.3%, while target-level FOV identification reached 97.7% consistency. The quantitative reconstruction performance of the Stokes parameters is summarized in Table 1, where the mean absolute errors (MAE) of the normalized Stokes components are reported. The results indicate high-fidelity polarization recovery after optical folding and intensity superposition.

To evaluate spatial discrimination capability, a confusion matrix was computed for the nine FOV regions. All regions achieved classification accuracies above 90%, demonstrating uniform separability in the Stokes domain. Minor misclassifications were mainly observed between polarization states with similar linear components, which is consistent with the theoretical proximity of these states in the polarization space.

The equivalent signal-to-noise ratio (SNR) is estimated based on the ratio between the mean signal intensity and noise variance, expressed as SNR = 10 log10 (S^2^/σ^2^). For the simulated noise levels, the corresponding SNR values range from approximately 40 dB to 26 dB, which are consistent with typical infrared imaging conditions under high signal-to-background contrast scenarios.

To further analyze robustness against measurement noise, additive Gaussian noise with standard deviation ranging from σ = 0 to 0.05 was introduced into the six-channel polarization measurements. The corresponding pixel-level and target-level classification accuracies are listed in Table 2. As the noise level increased, pixel-level accuracy gradually decreased from 97.3% to 86.8%. However, the target-level identification accuracy remained above 95% for σ ≤ 0.02, indicating stable reconstruction performance under moderate noise conditions.

In addition, sensitivity to polarization modulation uncertainty was investigated by introducing angular deviations to the predefined polarization parameters. Both the polarization azimuth angle (ψ) and ellipticity angle (χ) were perturbed within ±15°. The quantitative results are summarized in Table 3, showing that the classification accuracy remained above 92% when the modulation error was within ±10°, demonstrating strong tolerance to practical optical alignment and fabrication errors.

Overall, the quantitative results confirm that the proposed polarization multiplexing framework achieves high reconstruction accuracy while maintaining strong robustness against both measurement noise and modulation deviations, demonstrating its practical feasibility for compact wide-field infrared detection systems.

### 4.4. Comparative Performance Evaluation with Representative Methods

**Figure 6:**
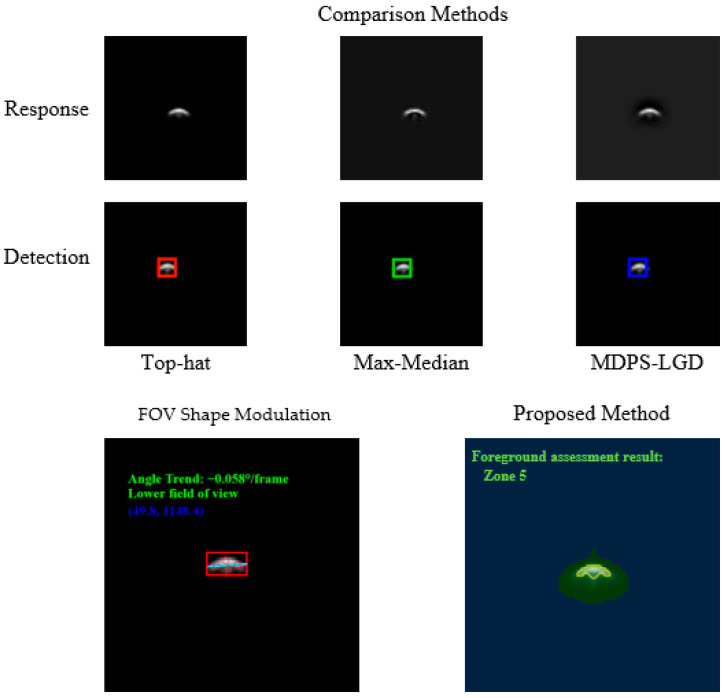
Comparison of small-target detection methods.

To further evaluate the effectiveness of the proposed polarization-encoded detector multiplexing method, we conducted a comparative analysis using the same aliased infrared dataset. The performance is compared against three traditional infrared small target (IRST) detection algorithms—Top-hat, Max-Median, and MDPS-LGD—as well as our previously proposed FOV shape modulation method [11]. Figure 6 shows the detection results of different algorithms.

All algorithms were evaluated on their ability to correctly identify the targets within aliased images. For the intensity-based traditional methods and the shape-modulation method, data from 10,800 frames were utilized. The results for the proposed polarization method are based on the target-level identification accuracy under a comparable noise level (σ=0.02).

As shown in Table 4, traditional IRST algorithms (Top-hat and Max-Median) exhibit relatively low detection rates when applied to multiplexed images. This is primarily because these methods rely solely on intensity and local contrast, which are severely degraded by the spatial aliasing and background superposition inherent in detector reuse. While the MDPS-LGD and FOV Shape Modulation methods improve performance by introducing morphological discriminators, they remain susceptible to target-background interference and point spread function (PSF) overlap.

In contrast, the proposed polarization-encoding framework achieves a superior target identification accuracy of 95.90%. The advantages are summarized as follows:

- Orthogonal Dimension: Unlike shape modulation which relies on geometric PSF deformation, polarization provides an orthogonal physical channel that is largely independent of the target’s spatial intensity distribution;
- Robustness to Aliasing: Even when multiple targets are completely overlapped in the intensity domain, their distinct Stokes vector signatures allow for high-fidelity separation;
- Signal-to-Noise Gain: The multi-channel polarization measurement (six-channel) provides redundant information that helps suppress random noise during the Stokes inversion process.

These results demonstrate that extending the modulation domain from scalar intensity to vector-field polarization significantly improves the reliability of target reconstruction in compact wide-field infrared sensing systems.

## 5. Discussion

The simulation results demonstrate that the proposed polarization-encoded FOV multiplexing framework achieves high reconstruction accuracy and strong robustness under various perturbation conditions. This section further discusses the underlying mechanisms, practical implications, and potential limitations of the proposed approach.

### 5.1. Physical Interpretation of Polarization-Domain Multiplexing

The effectiveness of the proposed method originates from the introduction of polarization as an additional physical encoding dimension. Unlike conventional spatial or morphological encoding approaches, which rely solely on geometric separation or point spread function variation, polarization encoding maps spatial information into the Stokes space.

Even after severe spatial aliasing caused by optical folding and intensity superposition, the polarization signatures associated with different FOV regions remain distinguishable. This orthogonality in the Stokes domain enables reliable separation of multiplexed signals and explains the high classification accuracy observed in the simulations.

Furthermore, the redundancy introduced by multi-channel polarization measurements improves noise tolerance. Since the Stokes parameters are reconstructed from multiple observations, random measurement perturbations are partially averaged during the inversion process, resulting in stable performance under moderate noise levels.

### 5.2. System-Level Advantages for Compact Infrared Imaging

From a system perspective, the proposed framework provides an effective solution for detector resource constraints in space-based or compact infrared imaging systems. By allowing multiple FOV regions to share a single detector sub-region, the method enables:

- Detector array size reduction;
- Lower cooling and power requirements;
- Reduced system volume and payload.

Compared with purely geometric multiplexing methods, polarization encoding does not require additional spatial separation structures, and therefore maintains system compactness while significantly improving separability.

Moreover, the simulation results indicate uniform discrimination performance across all nine FOV regions, suggesting good scalability for multi-channel wide-field detection scenarios.

### 5.3. Sensitivity to Practical Implementation Factors

Although the proposed method demonstrates strong robustness under simulated conditions, several practical factors may influence system performance in real infrared imaging scenarios.

First, the impact of background radiation and clutter should be considered. In realistic infrared search and track (IRST) systems, background signals originate from Earth emission, atmospheric scattering, and sensor noise. According to radiometric modeling results presented in our previous work, the background radiation power is several orders of magnitude lower than that of high-temperature targets. Therefore, detector multiplexing introduces only a limited increase in background energy, and the resulting signal-to-noise ratio degradation is relatively small (typically within several dB). This indicates that polarization-domain encoding does not significantly compromise target detectability under practical conditions.

Second, the noise model adopted in this study is based on additive Gaussian noise, which serves as a first-order approximation of detector noise. In practical infrared systems, photon noise and shot noise are dominant, following Poisson statistics. However, the proposed method relies primarily on polarization-state discrimination rather than intensity contrast, making it less sensitive to the exact noise distribution. Future work will incorporate photon noise modeling and experimentally validate system performance under realistic noise conditions.

Third, polarization multiplexing introduces an additional encoding dimension without fundamentally reducing the total collected optical energy. In an ideal system, the signal energy is preserved and redistributed across polarization channels, resulting in no intrinsic SNR loss. However, practical implementations may introduce additional losses due to imperfect polarization modulation, optical transmission inefficiencies, and multi-channel detection noise. Despite these factors, the redundancy provided by multi-channel Stokes reconstruction enhances robustness against noise perturbations.

Overall, the proposed method achieves a favorable trade-off between system compactness, information capacity, and noise robustness, demonstrating strong potential for practical deployment in compact wide-field infrared sensing systems.

Another practical consideration is the trade-off between temporal resolution and polarization measurement. Sequential acquisition of polarization states may introduce latency, especially when full Stokes parameters are required. However, this limitation can be mitigated by adopting polarization imaging sensors capable of parallel acquisition.

In scenarios where temporal resolution is critical, snapshot polarization imaging architectures should be preferred. Conversely, in applications where spatial resolution is not the dominant constraint, polarization cameras can be employed to directly capture multi-channel polarization information with reduced system complexity. This flexibility enables the proposed method to be adapted to different system-level requirements.

### 5.4. Limitations and Future Work

The current study is based on numerical simulations with ideal optical conditions. Several aspects remain to be further investigated.

First, experimental validation using a laboratory polarization imaging setup is required to verify real-system feasibility [26]. A feasible implementation can be realized by integrating a polarization modulation mask (e.g., micro-polarizer or waveplate array) at the intermediate image plane, combined with a folding optical system to achieve multi-FOV superposition. Polarization-resolved measurements can be performed either through sequential analyzer configurations or using a division-of-focal-plane polarization camera.

In scenarios where temporal resolution is critical, polarization cameras offer a practical solution for snapshot Stokes acquisition, albeit with reduced spatial resolution. Therefore, future system design will involve a trade-off between spatial resolution, temporal resolution, and polarization accuracy. Experimental validation under realistic infrared conditions will further confirm the feasibility and robustness of the proposed method.

Second, the current study assumes fully polarized modulation states to establish an ideal performance benchmark. However, in practical infrared imaging scenarios, the observed radiation is often partially polarized or even unpolarized. In such cases, the polarization components (S_1_, S_2_, S_3_) are reduced in magnitude, which may decrease the separability between different FOV channels in the Stokes domain. Nevertheless, since the proposed method relies on relative differences in polarization states rather than absolute polarization magnitude, discrimination can still be achieved when sufficient polarization contrast exists. Future work will focus on extending the framework to mixed-polarization conditions by incorporating degree-of-polarization (DoP) weighting and developing robust classification strategies under low-polarization environments.

Third, the present study focuses on FOV region identification. Future research will explore the integration of polarization-domain multiplexing with advanced detection algorithms for multi-target tracking and real-time processing. When multiple targets originating from different FOV regions overlap spatially on the detector, they may form a single connected region in the intensity image. In such cases, the majority-voting strategy may fail to correctly assign the FOV label, as the polarization information becomes mixed within the segmented region. This represents a fundamental challenge for the current framework. Future work will focus on addressing this limitation by incorporating temporal tracking, multi-target separation strategies, or advanced clustering methods in the polarization domain.

### 5.5. Summary

This paper proposes a polarization-encoded field-of-view multiplexing framework for wide-field infrared imaging systems under detector resource constraints. By introducing polarization as an additional encoding dimension, spatial information from multiple FOV regions is mapped into distinct Stokes vectors and subsequently reconstructed through polarization-domain decoding.

Simulation results demonstrate that the proposed method achieves a pixel-level classification accuracy of 97.3% under noise-free conditions, while maintaining target-level identification accuracy above 95% at a noise level of σ ≤ 0.02. The normalized Stokes reconstruction error is below 0.02, and classification accuracy remains higher than 93% under polarization modulation deviations within ±10°, indicating strong robustness against both noise and system imperfections.

Compared with conventional intensity-based and morphology-based multiplexing methods, the proposed approach extends the encoding dimension from scalar intensity to vector-field polarization, significantly enhancing separability and robustness. Furthermore, the method enables multi-FOV detection using a single detector, reducing system size, power consumption, and complexity.

These results demonstrate that polarization-domain multiplexing provides a physically interpretable and computationally efficient solution for compact wide-field infrared sensing systems, with promising application potential in space-based detection and surveillance scenarios.

## Figures and Tables

**Table 1:** Stokes reconstruction error (noise-free).

Component	MAE	RMSE
S_1_/S_0_	0.018	0.026
S_2_/S_0_	0.020	0.029
S_3_/S_0_	0.017	0.025

**Table 2:** Classification accuracy under noise.

Noise σ	Pixel Accuracy (%)	Target Accuracy (%)	SNR (dB)
0	97.3	97.7	∞
0.01	96.7	96.8	40
0.02	95.9	96.5	34
0.03	94.6	95.2	31
0.04	92.4	92.8	28
0.05	86.8	90.1	26

**Table 3:** Accuracy under polarization modulation error.

Angle Error (Deg)	Pixel Accuracy (%)
0	97.3
2	96.9
5	94.5
10	92.2
15	88.6

**Table 4:** Performance comparison of different discrimination methods.

Method	$P_{d}$ (%)	$P_{fa}$ (%)
Top-hat	74.82	3.51
Max-Median	68.59	4.87
MDPS-LGD	89.63	2.52
FOV Shape Modulation	94.04	1.80
Proposed Polarization Method	95.90	1.25

## Data Availability

The data presented in this study are available on request from the corresponding author.
